# Mechanistic Modulation of Autophagy by Bioactive Natural Products: Implications for Human Aging and Longevity

**DOI:** 10.3390/nu18050863

**Published:** 2026-03-07

**Authors:** Maroua Jalouli, Abdel Halim Harrath, Mohammed Al-Zharani, Md Ataur Rahman

**Affiliations:** 1 Department of Biology, College of Science, Imam Mohammad Ibn Saud Islamic University (IMSIU), Riyadh 11623, Saudi Arabia; mejalouli@imamu.edu.sa (M.J.); mmylzahrani@imamu.edu.sa (M.A.-Z.); 2 Department of Zoology, College of Science, King Saud University, Riyadh 11451, Saudi Arabia; hharrath@ksu.edu.sa; 3 Department of Oncology, Karmanos Cancer Institute, Wayne State University, Detroit, MI 48201, USA

**Keywords:** bioactive natural products, autophagy modulation, human aging, longevity, oxidative stress, cellular homeostasis

## Abstract

Autophagy is an evolutionarily preserved intracellular degradation process pivotal in maintaining proteostasis, mitochondrial homeostasis, and metabolic equilibrium, all of which are dysregulated with aging. Aberrant autophagy has been recognized as a hallmark of human aging and age-related diseases, including neurodegeneration, metabolic dysfunction, cardiovascular diseases, and cancer. Bioactive natural compounds derived from plants, foods, and marine organisms have emerged as potent modulators of autophagy, offering a promising strategy to counteract aging and promote healthy lifespan. Mechanistically, these compounds regulate autophagy by modulating key signaling pathways, such as AMPK, PI3K/AKT/mTOR, SIRT1, and FOXO, while also alleviating oxidative stress, inflammation, and mitochondrial dysfunction. Natural compounds like polyphenols, flavonoids, alkaloids, terpenoids, and carotenoids exhibit dual roles by restoring age-related suppressed autophagic flux and inhibiting excessive autophagy-induced cell death. In this review, we provide a comprehensive overview of the molecular mechanisms through which bioactive natural compounds modulate autophagy and impact human aging and longevity. We discuss both experimental and clinical evidence supporting their geroprotective effects, limitations regarding bioavailability and dose-dependent effects, and prospects for the utilization of autophagy-targeting natural products in aging intervention strategies.

## 1. Introduction

Autophagy is a lysosome-dependent catabolic process that degrades damaged organelles, misfolded proteins, and toxic aggregates, thus maintaining metabolic homeostasis and cellular integrity throughout the lifespan [1]. Aging is a multifactorial biological process characterized by a gradual decline in cellular, tissue, and organ function, eventually leading to an increased risk of chronic diseases and death [2]. At the cellular level, aging is driven by the accumulation of molecular damage, impaired protein quality control, mitochondrial dysfunction, oxidative stress, and chronic low-grade inflammation [3]. Evidence from various sources supports the view that autophagic flux declines with age in many tissues, including the brain, liver, skeletal muscle, and cardiovascular system [4]. Age-related impairment in autophagy leads to cellular senescence, decreased stress resilience, and functional decline, all of which accelerate the aging process and contribute to the development of age-related diseases [5]. Experimental studies in model organisms have revealed that genetic or pharmacological activation of autophagy can extend lifespan and promote health span, suggesting autophagy as a therapeutic target for aging intervention [6]. Consequently, strategies to restore or optimize autophagic flux have gained significant attention in the field of aging research.

In recent decades, bioactive natural compounds from food sources, medicinal plants, and marine organisms have emerged as powerful and physiologically relevant modulators of autophagy [7]. These molecules often exhibit multitargeted actions that are closely aligned with the biological complexity of aging. Generally, bioactive natural compounds act by reestablishing dysfunctional autophagic processes, thus modulating cellular resilience to age-related stress rather than inducing excessive autophagy [8]. Polyphenols, flavonoids, alkaloids, terpenoids, and carotenoids are known to regulate autophagy by modulating the activity of key signaling pathways, such as AMPK, PI3K/AKT/mTOR, SIRT1, and FOXO, which are also crucial in the regulation of aging and lifespan [9].

In addition to their autophagy-modulating properties, natural products possess other geroprotective effects, including the reduction in oxidative stress, inhibition of chronic inflammation, improvement of mitochondrial quality control, and maintenance of metabolic homeostasis [10]. These coordinated effects position bioactive natural compounds as promising agents for promoting healthy aging and delaying the onset of age-related diseases. However, despite promising experimental data, several challenges still need to be addressed for translating these molecules into clinically relevant anti-aging interventions, including bioavailability, dosage specificity, and tissue-targeted effects. In this review, we explore the molecular mechanisms by which bioactive natural compounds regulate autophagy and their implications for human aging and longevity. By integrating molecular, experimental, and emerging clinical evidence, we aim to provide a comprehensive framework to better understand how the regulation of autophagy by natural products can be leveraged to promote healthy aging and extend lifespan.

## 2. Molecular Mechanisms of Autophagy in Human Aging and Longevity Control

**Figure 1:**
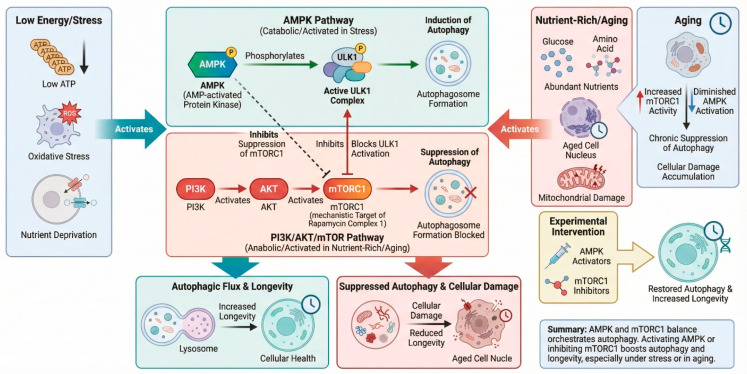
**Evolutionarily conserved signaling mechanisms regulated by autophagy.** Low energy, oxidative stress, or food deprivation activates AMPK by decreasing ATP and increasing cellular stress signals. Activated AMPK phosphorylates ULK1, forming the active complex and autophagosomes. AMPK also reduces mTORC1 activity, releasing autophagy inhibitory effects. Nutrient-rich circumstances and aging promote PI3K/AKT signaling and mTORC1 activation. ULK1 activation and autophagosome formation are blocked by active mTORC1, suppressing autophagic flow. Age-related chronic mTORC1 activation reduces AMPK signaling, mitochondrial malfunction, protein and organelle degradation, and cellular damage. AMPK-mTORC1 balance as a major autophagy regulator. Experimental therapies like AMPK activators and mTORC1 inhibitors increase autophagic flux, lysosomal function, mitochondrial quality control, and cellular health. These conserved signaling pathways regulate autophagy, cellular homeostasis, and longevity across species by integrating energy status, redox balance, and nutrient sensing.

Autophagy is a highly regulated, lysosome-dependent catabolic process essential for maintaining cellular quality control and adaptive stress responses, both of which are key determinants of human aging and longevity. At the molecular level, autophagy is induced in response to nutrient deprivation, oxidative stress, and metabolic imbalances through the intricate orchestration of evolutionarily conserved signaling pathways [11]. Central to its regulation is the balance between anabolic and catabolic signals, with the AMPK and PI3K/AKT/mTOR signaling pathways playing a dominant role [12]. AMP-activated protein kinase (AMPK) is an energy sensor that triggers autophagy through the phosphorylation of ULK1 and the inhibition of mTORC1 activity under low-energy conditions [13]. On the other hand, mTORC1 acts as a negative regulator of autophagy by blocking the activation of the ULK1 complex under nutrient-rich conditions. Dysregulation of autophagy is associated with the aging process, including persistent activation of mTORC1 and blunted AMPK signaling, leading to chronic suppression of autophagy and the accumulation of damaged cellular components [14]. Reinstating AMPK activity or inhibiting mTOR signaling has been shown to enhance autophagic flux and extend lifespan in various model systems. Figure 1 explores the evolutionarily conserved nutrient-sensing and stress–response pathways that regulate autophagy and influence aging and longevity.

**Figure 2:**
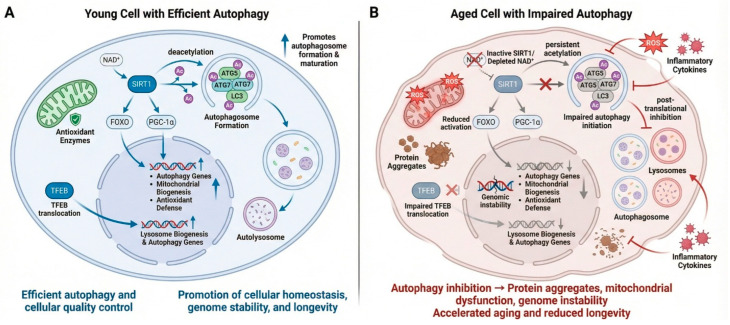
**Sirtuin-mediated regulation of autophagy and longevity in molecular interconnections and aging impairments.** (**A**) **Young Cell with Efficient** Autophagy: In young cells, sufficient NAD^+^ levels support SIRT1 activity, leading to deacetylation of autophagy-related proteins such ATG5, ATG7, and LC3. A post-translational change boosts autophagosome production and maturation. SIRT1 upregulates genes involved in autophagy, mitochondrial biogenesis, and antioxidant defense by activating transcriptional regulators such FOXO and PGC-1α. While translocating to the nucleus, TFEB stimulates lysosomal biogenesis and autophagy gene expression, promoting autophagic flux. These coordinated molecular interactions preserve mitochondrial quality, redox balance, genomic stability, and cellular health, promoting longevity. (**B**) **Aged Cell with Impaired Autophagy**: As we age, NAD^+^ depletion reduces SIRT1 activity, causing autophagy-related protein acetylation and decreased autophagy initiation. Decreased FOXO, PGC-1α, and TFEB activation reduces autophagy, lysosomal, and antioxidant gene expression. Protein clumps, reactive oxygen species, mitochondrial malfunction, and genetic instability further reduce autophagic flow. Chronic inflammation and post-translational inhibition worsen lysosomal dysfunction. These molecular disruptions cause cellular quality control issues, faster aging, and shorter lifespans.

The Sirtuin family of proteins, particularly SIRT1, modulates longevity by deacetylating key autophagy-related proteins, including ATG5, ATG7, and LC3, thereby promoting the formation and maturation of autophagosomes [15]. SIRT1 also regulates transcription factors like FOXO and PGC-1α, which coordinate autophagy, mitochondrial biogenesis, and antioxidant defense pathways [16]. The age-related decline in NAD^+^ levels impairs SIRT1 activity, impairing autophagy and accelerating cellular aging. Transcription factor EB (TFEB) is a master regulator of lysosomal biogenesis and autophagy-related gene expression at the transcriptional level [17]. The reduced nuclear translocation of TFEB with age impairs lysosomal function and autophagic degradation [18]. Additionally, an excess of reactive oxygen species and chronic inflammation can inhibit autophagy through post-translational modifications of autophagy-related proteins. Collectively, the dysregulation of autophagy results in the accumulation of protein aggregates, dysfunctional mitochondria, and genomic instability, all of which contribute to aging and limit lifespan. Targeting these molecular checkpoints to restore balanced autophagic activity is a promising strategy to promote healthy aging and extend longevity (Figure 2).

Figure 1: **The central molecular pathways regulate autophagy and their impact on human aging and longevity**. The AMPK pathway serves as a principal autophagy enhancer in low-energy settings by detecting increased AMP levels and activating the ULK1 complex to commence autophagosome production. The mTORC1 pathway serves as a principal inhibitor of autophagy, combining signals from nutrients, glucose, amino acids, and insulin through PI3K-AKT signaling to decrease ULK1 activation in nutrient-abundant environments. Sirtuins and NAD^+^ signaling, especially SIRT1, enhance autophagy by deacetylating essential autophagy proteins ATG5, ATG7, and LC3, while also facilitating mitochondrial homeostasis, antioxidant defenses, and longevity-related transcriptional programs via FOXO and PGC-1α. The reduction of NAD^+^ with age restricts these protective benefits. TFEB-mediated lysosomal regulation governs the transcription of autophagy and lysosomal genes, augmenting lysosome formation and degradative capacity vital for effective autophagic flux. Inhibitory factors, such as increased reactive oxygen species and chronic inflammation, hinder the functionality of autophagy proteins and lysosomal breakdown. The coordinated regulation of these pathways maintains cellular homeostasis, mitigates aging-related damage, and promotes longevity.

## 3. Bioactive Natural Products as Autophagy Modulators in Aging and Longevity Control

**Figure 3:**
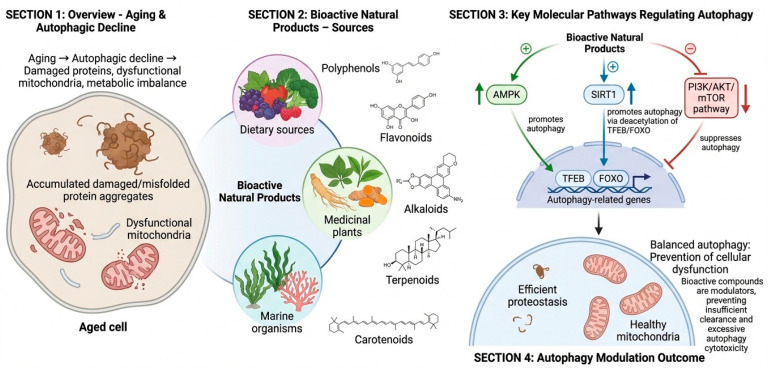
**Modulation of autophagy in aging by bioactive natural products**. Section 1 demonstrates the age-associated reduction in autophagy, resulting in the accumulation of damaged or misfolded protein aggregates, mitochondrial malfunction, and metabolic dysregulation in older cells. Section 2 delineates principal sources and categories of bioactive natural compounds, encompassing dietary polyphenols and flavonoids, plant-derived alkaloids and terpenoids, and marine-derived carotenoids, all of which jointly influence autophagy control. Section 3 illustrates the principal molecular pathways influenced by these substances, wherein the activation of AMPK and SIRT1 promotes autophagy via phosphorylation and deacetylation processes, while the inhibition of the PI3K/AKT/mTOR pathway alleviates restrictions on autophagic signaling. Transcriptional regulation by TFEB and FOXO enhances the expression of genes associated with autophagy and lysosomes, hence reinstating autophagic flow. Section 4 illustrates the results of balanced autophagy modulation, encompassing enhanced proteostasis, preservation of healthy mitochondria, and avoidance of cellular malfunction.

Bioactive natural compounds have emerged as attractive candidates for autophagy modulation with implications for human aging and lifespan regulation [8]. Unlike conventional single-target synthetic drugs, natural products often exhibit pleiotropic actions that align with the complex and multifactorial nature of aging. Their ability to modulate autophagy contextually, restoring impaired autophagic flux rather than causing unregulated induction of autophagy, positions them as potential candidates for promoting healthy aging and lifespan extension [19]. Decreased autophagy is a hallmark of aging, resulting in the accumulation of damaged proteins, dysfunctional mitochondria, and metabolic disturbances [4]. Bioactive natural compounds counteract these changes by fine-tuning key molecular pathways involved in autophagy regulation, including AMPK, PI3K/AKT/mTOR, SIRT1, FOXO, and TFEB [20]. These compounds act primarily as modulators of autophagy rather than as strong activators, avoiding both insufficient clearance and excessive cytotoxicity associated with dysregulated autophagy. Major groups of compounds involved in autophagy modulation include polyphenols, flavonoids, alkaloids, terpenoids, and carotenoids, which are found in dietary components, medicinal plants, and marine organisms (Figure 3).

Polyphenols represent one of the most extensively studied groups of natural compounds with autophagy modulatory effects (Table 1). Polyphenols, such as resveratrol, curcumin, epigallocatechin gallate, and quercetin, are abundant in fruits, vegetables, tea, coffee, and red wine, and have been shown to significantly impact cellular aging pathways [21]. Mechanistically, polyphenols enhance AMPK and SIRT1 signaling, while also inhibiting mTORC1 activity, thus promoting balanced autophagy induction and autophagic flux [22]. Additionally, their antioxidants and anti-inflammatory properties help reduce oxidative and inflammatory stress, which can otherwise impair autophagy during aging. The combined effects of polyphenols promote mitochondrial quality control, proteostasis, and metabolic flexibility, contributing to improved cellular resilience and longevity [23].

Resveratrol, a stilbene polyphenol, primarily acts as a chain-breaking radical-trapping antioxidant. The activity encompasses hydrogen atom transfer and successive proton-loss electron transfer processes, enabled by phenolic hydroxyl groups [24]. Its activity is affected by solvent polarity and pH, exhibiting increased reactivity under slightly alkaline conditions. With moderate lipophilicity, it divides into membranes, scavenging peroxyl radicals and modulating superoxide-derived species [25]. While not a traditional enzymatic antioxidant, it enhances the expression of endogenous ROS-detoxifying enzymes and indirectly facilitates the regeneration of glutathione and α-tocopherol through Nrf2 activation [26]. Curcumin, a diarylheptanoid, functions as a radical-scavenging and metal-chelating antioxidant. It experiences hydrogen atom donation and electron transfer, with keto-enol tautomerism affecting solvent-dependent reactivity [27]. Its lipophilicity promotes membrane localization and the neutralization of lipid peroxyl radicals. Curcumin additionally augments glutathione biosynthesis and bolsters redox enzyme systems [28]. EGCG, quercetin, fisetin, kaempferol, apigenin, luteolin, genistein, and chlorogenic acid are derivatives of flavonoids or phenolic acids that primarily act as radical-trapping antioxidants via hydrogen atom transfer and electron transfer pathways, influenced by hydroxyl patterns and ring conjugation [29,30]. Their reactivity fluctuates with pH and polarity, affecting the scavenging of superoxide, hydroxyl, and nitrogen-centered radicals. Various chelate transition metals indirectly promote the regeneration of glutathione, coenzyme Q10, and α-tocopherol through redox cycling networks [31].

**Table 1:** Major Polyphenols and their autophagy modulation in aging and longevity regulation.

Polyphenol Compound	Major Sources	Autophagy-Modulating Mechanism	Relevance to Aging and Longevity Control	Ref.
Resveratrol	Grapes, berries, peanuts, red wine	Activates SIRT1 and supports AMPK signaling, promotes autophagy and stress resistance pathways	Linked to longevity-related signaling (SIRT1 activation) and autophagy-mediated lifespan extension in model systems	[32]
Curcumin	Turmeric (*Curcuma longa*)	Promotes autophagy via SIRT1, AMPK, and mTOR suppression, reduces ROS	Demonstrated anti-senescence and anti-aging effects in experimental aging settings through autophagy restoration	[33]
Epigallocatechin-3-gallate (EGCG)	Green tea	Shifts balance toward autophagy by modulating mTOR–AMPK signaling and requiring ULK1	Reported to support cellular stress adaptation via autophagy, with evidence in vascular aging contexts	[34]
Quercetin	Onions, apples, berries, capers, leafy greens	Activates AMPK/SIRT1-linked protective signaling and can promote SIRT1-dependent autophagy	Implicated in counteracting stress and degenerative processes where autophagy and senescence are involved	[35]
Fisetin	Strawberries, apples, persimmons, onions	Can suppress mTOR signaling components and induce autophagy, also linked to AMPK/SIRT1 and inflammation control	Studied as a senotherapeutic, associated with improved healthspan and lifespan in animal models, mechanistically tied to autophagy pathways	[36]
Kaempferol	Kale, spinach, broccoli, tea, berries	Activates AMPK, promotes ULK1 phosphorylation, inhibits mTORC1, induces autophagy	Supports cellular quality control programs relevant to aging biology through AMPK–mTOR autophagy regulation	[37]
Apigenin	Parsley, celery, chamomile, citrus	Induces autophagy via AMPK activation and mTOR inhibition, also reported via PI3K/AKT/mTOR suppression	Highlights how dietary flavones can restore autophagic signaling relevant to aging-associated proteostasis decline	[38]
Luteolin	Celery, green pepper, thyme, chamomile	Promotes autophagy through AMPK/mTOR-linked pathways (context-dependent), supports stress adaptation	Relevant to longevity control by reinforcing homeostatic pathways that decline with age, including autophagy regulation	[39]
Genistein	Soybeans, soy foods	Induces autophagy via mTOR pathway modulation, supports degradative clearance mechanisms	Used in disease models where enhanced autophagy is beneficial, relevant to aging due to autophagy’s role in proteostasis maintenance	[40]
Chlorogenic acid	Coffee, apples, pears, blueberries, artichoke	Activates autophagy via AMPK/mTOR/ULK1 signaling and can intersect with lysosomal regulation	Links dietary phenolic acids with autophagy control under stress conditions that overlap with aging biology	[41]

Flavonoids, a subclass of polyphenols, encompass compounds like luteolin, apigenin, kaempferol, and myricetin, which are found in fruits, vegetables, and medicinal herbs [42]. Flavonoids regulate autophagy by modulating both upstream signaling pathways and the transcriptional regulation of autophagy-related genes [43]. Many flavonoids facilitate AMPK activation and inhibit PI3K/AKT signaling, leading to mTOR inhibition and controlled induction of autophagy [44]. Simultaneously, flavonoids regulate FOXO and TFEB transcription factors, promoting lysosomal biogenesis and maintaining efficient autophagic degradation [17]. These effects are particularly relevant in aging tissues, such as the brain and skeletal muscle, where autophagy dysfunction contributes to functional decline. Table 2 presents the predominant flavonoids and their modulation of autophagy in the regulation of aging and longevity.

Flavonoids including luteolin, apigenin, kaempferol, myricetin, fisetin, quercetin, naringenin, hesperidin, baicalein, and genistein are polyphenolic radical-trapping antioxidants (RTAs) distinguished by phenolic hydroxyl groups that facilitate hydrogen atom transfer and single-electron transfer mechanisms [45]. Their antioxidant activity often occurs through hydrogen atom transfer, consecutive proton-loss electron transfer, or single-electron transfer followed by proton transfer, contingent upon hydroxyl substitution patterns and conjugation across the C ring [46]. Ortho-dihydroxy structures, especially in quercetin, luteolin, and baicalein, augment the stability of phenoxyl radicals via resonance, hence enhancing the efficacy in scavenging peroxyl (ROO•), superoxide (O2•^−^), hydroxyl (•OH), and nitrogen-centered radicals such as •NO and •NO_2_ [47]. Their reactivity is contingent upon pH, as deprotonation under somewhat alkaline conditions amplifies electron-donating capacity [48]. The polarity of solvents affects the stability of radicals and their bioavailability. Lipophilicity differs: flavonols like quercetin and kaempferol demonstrate partial membrane partitioning, facilitating the prevention of lipid peroxidation, while glycosylated hesperidin is more hydrophilic and mostly functions in aqueous environments [49]. In addition to direct radical scavenging, certain flavonoids enhance the expression of endogenous antioxidant enzymes such as superoxide dismutase, catalase, and glutathione peroxidase through Nrf2 activation [50]. Numerous individuals engage indirectly in the breakdown of enzymatic ROS/RNS by maintaining glutathione reserves and promoting redox cycling. Quercetin and luteolin can restore α-tocopherol in lipid environments, whereas other compounds facilitate the regeneration of glutathione, coenzyme Q10, and lipoic acid redox states [51].

**Table 2:** Principal flavonoids and their modulation of autophagy in the regulation of aging and longevity.

Flavonoid Compound	Major Dietary or Botanical Sources	Autophagy-Modulating Mechanisms	Relevance to Aging and Longevity Control	Ref.
Luteolin (flavone)	Celery, green pepper, parsley, thyme, chamomile	Modulates AMPK-dependent autophagy, supports restoration of autophagic flux under stress	Supports proteostasis and stress resilience, relevant to tissues vulnerable in aging	[9]
Apigenin (flavone)	Parsley, celery, chamomile, citrus	Promotes autophagy via AMPK activation, ULK1 phosphorylation, mTOR suppression, also inhibits PI3K/AKT/mTOR	Helps rebalance autophagy in stress conditions, potentially limiting age-related dysfunction	[52]
Kaempferol (flavonol)	Kale, spinach, broccoli, tea, berries	Activates AMPK, induces ULK1 phosphorylation, inhibits mTORC1, increases LC3 processing	Enhances cellular quality control and mitochondrial turnover, processes that decline with age	[37]
Myricetin (flavonol)	Berries, grapes, tea, walnuts, vegetables	Induces autophagy by inhibiting PI3K/AKT/mTOR or directly suppressing mTOR signaling (context-dependent)	Supports autophagy restoration under metabolic and oxidative stress linked to aging biology	[53]
Fisetin (flavonol)	Strawberries, apples, onions, persimmons	Stimulates autophagy with TFEB activation, likely via mTORC1 inhibition, also engages stress response programs	Relevant to healthy aging concepts due to autophagy-lysosome reinforcement and cellular cleanup	[54]
Quercetin (flavonol)	Onions, apples, berries, capers, leafy greens	Promotes TFEB nuclear translocation and enhances TFEB activity, reported as a direct mTOR inhibitor in some systems	Strengthens lysosome biogenesis and autophagic clearance, key for proteostasis in aging	[55]
Naringenin (flavanone)	Citrus fruits (grapefruit, oranges), tomatoes	Enhances autophagy through AMPK–AKT/mTOR and AMPK/mTOR/ULK1-linked regulation (model dependent)	Supports autophagy under inflammatory and oxidative conditions relevant to age-associated decline	[56]
Hesperidin (flavanone glycoside)	Citrus peel and pulp (orange, lemon)	Reported to stimulate autophagy coupled with PI3K/AKT/mTOR axis modulation in disease models	Highlights dietary flavanones as autophagy modulators that may support resilience during aging	[57]
Baicalein (flavone)	*Scutellaria baicalensis* (Chinese skullcap)	Activates mitochondrial autophagy via SIRT1/AMPK/mTOR pathway modulation	Especially relevant to brain aging concepts because mitophagy supports neuronal health	[58]
Genistein (isoflavone)	Soybeans, soy foods	Reported to induce autophagy through mTOR inactivation and autophagy-dependent mechanisms in various models	Supports the concept of diet-derived isoflavones influencing longevity pathways via autophagy	[40]

Alkaloids, a diverse class of nitrogen-containing compounds, are widely distributed in plants, many of which possess medicinal properties and play a significant role in regulating aging through autophagy [59]. Alkaloids modulate aging-related autophagy chiefly by adjusting the AMPK-mTOR pathway, altering stress factors like ROS and inflammation, and modulating reforming transcriptional or epigenetic mechanisms that govern autophagy gene expression [60]. Significantly, many alkaloids seem to normalize autophagic flux instead of only enhancing autophagy, a characteristic that is mechanistically associated with good aging and longevity regulation [61]. Alkaloids and related nitrogenous compounds such as berberine, caffeine, and spermidine have been shown to modulate lifespan pathways by enhancing autophagic capacity. Spermidine has garnered attention for its ability to induce autophagy through epigenetic regulation, including inhibition of histone acetyltransferases and activation of autophagy gene expression [62]. Berberine activates AMPK signaling, improves mitochondrial function, and reduces chronic inflammation, indirectly promoting autophagy during aging [63]. These properties position alkaloids as regulators of autophagy and metabolic health. Important alkaloids or alkaloid-like polyamines used in aging control via autophagy modulation are presented in Table 3.

Spermidine, a polyamine rather than a phenolic antioxidant, is generally categorized as a preventative redox modulator [64]. It does not operate as a radical-trapping antioxidant via hydrogen atom transfer. Its biological effects stem from electrostatic interactions with nucleic acids and proteins, modification of acetyl groups, and epigenetic regulation of autophagy genes [65]. Its solubility in water facilitates distribution within the cytosol and nucleus. Spermidine indirectly diminishes ROS via improving mitochondrial quality control and facilitating autophagic clearance, rather than directly neutralizing superoxide, hydroxyl radicals, or nitrogen radicals [66]. It facilitates glutathione homeostasis through enhanced cellular turnover. Berberine, tetrandrine, matrine, harmine, piperine, capsaicin, evodiamine, and galantamine are heterocyclic alkaloids characterized by considerable lipophilicity [67]. They are more accurately classified as preventative antioxidants and redox-modulating signaling agents instead of traditional radical-trapping antioxidants [68]. Their processes entail the control of mitochondrial reactive oxygen species generation, activation of AMPK, and inhibition of mTOR, rather than direct hydrogen atom transfer chemistry [69]. Their radical scavenging ability is constrained relative to polyphenols; however, some can moderately diminish superoxide and nitric oxide concentrations in aqueous settings. The polarity of solvents affects membrane permeability and mitochondrial localization, especially for lipophilic compounds such as capsaicin and piperine [70]. These alkaloids typically contribute to redox homeostasis indirectly by enhancing the expression of endogenous antioxidant enzymes, including superoxide dismutase, catalase, and glutathione peroxidase [71]. Multiple agents augment the restoration of glutathione levels and facilitate redox cycling mechanisms involving coenzyme Q10 and lipoic acid, whereas the protective effects on α-tocopherol are subordinate to membrane stabilization [72].

**Table 3:** Alkaloid chemicals that serve as autophagy modulators relevant to human aging biology and longevity regulation.

Alkaloid (or Alkaloid-like Polyamine)	Major Sources	Autophagy-Modulating Mechanisms	Relevance to Aging and Longevity Control	Ref.
Spermidine (polyamine)	Soybeans, wheat germ, mushrooms, aged cheese, fermented foods	Induces autophagy via epigenetic deacetylation programs, including histone acetyltransferase inhibition, upregulates autophagy genes and promotes cytoprotective autophagy	Reported lifespan extension in multiple model organisms in an autophagy-dependent manner, widely discussed as a geroprotective nutrient	[73]
Berberine	*Berberis* spp., *Coptis chinensis* (Rhizoma Coptidis)	Activates AMPK, suppresses mTOR and can engage ULK1, improves mitochondrial function and reduces inflammatory stress while enhancing autophagy	Links metabolic health and “healthy aging” pathways through AMPK, autophagy, and mitochondrial quality control, evidence is largely preclinical	[74]
Caffeine	Coffee, tea, cacao	Induces autophagy in vivo and in cells, reported mechanisms include Ca^2+^-AMPK signaling and AMPK/SIRT1 axis depending on tissue context	Relevant to longevity biology because enhanced autophagy and reduced mTOR signaling are associated with improved stress resistance and metabolic aging phenotypes	[75]
Tetrandrine (bisbenzylisoquinoline)	*Stephania tetrandra*	Induces autophagy via mTOR inactivation (AMPK-independent in some systems), increases autophagic flux in multiple cell models	Demonstrates strong autophagy pathway engagement, translational relevance for aging is indirect (via proteostasis and cellular cleanup mechanisms)	[76]
Matrine	*Sophora flavescens*	Induces autophagy via ROS–AMPK–mTOR axis and p53/AMPK signaling, with context-dependent pro-death versus protective autophagy	Highlights dose and context specificity, important for aging where excessive autophagy can be harmful, mechanistic relevance via AMPK–mTOR control	[77]
Harmine (β-carboline)	*Peganum harmala*, *Banisteriopsis caapi*	Promotes autophagy, commonly linked to Akt/mTOR inhibition, can increase LC3 and Beclin-1 in several models	Demonstrates autophagy engagement through nutrient signaling checkpoints relevant to aging, translational relevance remains mostly preclinical	[78]
Piperine	Black pepper (*Piper nigrum*)	Induces autophagy by mTORC1 inhibition, reported via PP2A activation and/or Akt/mTOR suppression, can enhance autophagic clearance in neuronal contexts	Relevant to aging phenotypes through support of proteostasis and stress adaptation, with mechanistic links to AMPK–mTOR balance and inflammation	[79]
Capsaicin (capsaicinoid alkaloid/vanilloid)	Chili peppers (*Capsicum* spp.)	Modulates autophagy through AMPK activation and Akt/mTOR inhibition, effects can vary by model and dose, sometimes affecting flux	Mechanistically intersects with canonical longevity nodes (AMPK, mTOR), highlights the importance of balanced autophagy for healthy aging	[80]
Evodiamine	*Evodia rutaecarpa* (Wu Zhu Yu)	Reported to regulate autophagy via p53/mTOR and intersects with AMPK–SIRT1 pathways in some contexts	Relevant as an autophagy modulator linked to oxidative stress control and cellular homeostasis, evidence is preclinical	[81]
Galantamine	*Galanthus* spp. (snowdrop), *Narcissus* spp.	Modulates autophagy under stress conditions, reported to inhibit Aβ-induced cytostatic autophagy while reducing ROS, indicating “flux normalization” rather than simple induction	Supports the concept that longevity-relevant benefit may come from restoring autophagy balance and reducing oxidative stress in brain aging contexts	[82]

Terpenoids, including monoterpenes, diterpenes, and triterpenes, are derived from aromatic plants, herbs, and marine organisms [83]. Compounds like ursolic acid, ginsenosides, and andrographolide have been found to modulate autophagy through regulation of mTOR, AMPK, and oxidative stress pathways [84]. Terpenoids often exhibit significant anti-inflammatory properties, which are crucial for maintaining autophagic function in the context of age-related chronic inflammation [85]. Terpenoids indirectly support lysosomal function and autophagic flux by reducing pro-inflammatory cytokine signaling and oxidative damage, thereby promoting tissue homeostasis and longevity [86]. Terpenoids as autophagy modulators essential to human aging biology and longevity regulation, emphasizing AMPK, PI3K/AKT/mTOR, ULK1, TFEB, lysosomal biogenesis, oxidative stress, and inflammation (Table 4).

Terpenoids, including ursolic acid, oleanolic acid, andrographolide, celastrol, carnosic acid, carnosol, dihydroartemisinin, artemisinin, triptolide, ginsenoside Rb1, and ginkgolide B, fundamentally vary from traditional phenolic radical-trapping antioxidants [87]. The majority are more accurately categorized as preventative antioxidants and redox-modulating electrophilic signaling molecules, rather than as direct hydrogen atom-donating antioxidants [88]. Their chemical architectures, characterized by conjugated double bonds, quinone methide groups, lactones, or triterpenoid backbones, promote the regulation of redox-sensitive signaling proteins instead of immediate radical scavenging [89]. Reaction methods generally encompass Michael addition or redox-sensitive cysteine interactions within Keap1, AMPK-regulatory complexes, or other thiol-containing proteins, hence indirectly modulating ROS generation and the expression of antioxidant enzymes [90]. In most triterpenoids, direct sequential proton-loss electron transfer pathways are negligible, unless in the presence of phenolic moieties, as observed in carnosic acid and carnosol, which exhibit traditional hydrogen atom-donating capabilities and lipid radical-trapping qualities [91].

The properties and polarity of solvents significantly affect their biological behavior. Numerous terpenoids exhibit significant lipophilicity, facilitating membrane partitioning and mitochondrial localization [92]. This lipophilicity facilitates the suppression of lipid peroxidation and the stabilization of membrane-associated oxidative processes; however, it constrains aqueous radical scavenging [93]. Instead of directly neutralizing O2•^−^, •OH, or nitrogen radicals, terpenoids primarily mitigate oxidative stress by stimulating endogenous antioxidant mechanisms, such as superoxide dismutase, catalase, glutathione peroxidase, and Nrf2-regulated enzymes [94]. Various compounds, notably carnosic acid and celastrol, aid in the preservation of glutathione redox equilibrium and indirectly promote the regeneration of α-tocopherol and coenzyme Q10 by sustaining overall cellular reducing capacity [95].

**Table 4:** Terpenoids as autophagy modulators related to human aging biology and longevity regulation.

Terpenoid (Class)	Major Sources	Autophagy Modulation Mechanism	Aging and Longevity Relevance	Ref.
Ursolic acid (triterpenoid)	Apple peel, rosemary, basil, oregano, cranberries	Induces autophagy via CaMKK–AMPK–mTOR signaling, can involve ROS and ER stress pathways, supports autophagic flux	Reinforces proteostasis and mitochondrial quality control, mechanisms linked to healthier aging trajectories	[96]
Oleanolic acid (triterpenoid)	Olive oil, *Ligustrum*, many medicinal plants	Activates AMPK, suppresses mTORC1, engages AMPK/mTOR/autophagy signaling, context-dependent protective vs. cytotoxic autophagy	Autophagy pathway intersects with metabolic resilience and inflammaging control, relevant to aging biology	[97]
Andrographolide (diterpenoid lactone)	*Andrographis paniculata* (medicinal herb)	Modulates autophagy through PI3K/Akt/mTOR inhibition and autophagy activation in several models	Autophagy-related stress adaptation and anti-inflammatory actions may support tissue homeostasis during aging	[98]
Celastrol (triterpenoid quinone methide)	*Tripterygium wilfordii* (Thunder god vine)	Potent TFEB activator, enhances autophagy and lysosomal biogenesis, increases lysosomal markers (e.g., LAMP1)	Enhances clearance of aggregation-prone proteins, relevant to brain aging and proteostasis decline	
Carnosic acid (diterpenoid)	Rosemary (*Rosmarinus officinalis*), sage	Activates AMPK and inhibits mTOR, autophagy contributes to neuroprotection and stress resilience	Mechanistically relevant to neuro-aging because improved autophagy supports mitochondrial quality and protein clearance	[99]
Carnosol (diterpenoid)	Rosemary, sage	Linked to AMPK activation and autophagy-related cytoprotection in injury models, supports stress-adaptive signaling	Anti-inflammatory and redox control can indirectly preserve lysosomal function and autophagic flux in aging-like stress	[100]
Dihydroartemisinin (DHA) (sesquiterpene lactone derivative)	Derived from *Artemisia annua* (sweet wormwood)	Induces autophagy via AMPK/mTOR regulation, reported autophagy-dependent mechanisms in senescent cells	Direct relevance to aging biology through reported clearance of senescent cells using autophagy-linked mechanisms	[101]
Artemisinin (sesquiterpene lactone)	*Artemisia annua*	Promotes (mitochondrial) autophagy and inhibits PI3K/AKT/mTOR in disease models, restores autophagy pathways	Relevant to longevity control via autophagy support in chronic inflammatory and metabolic disease contexts	[102]
Triptolide (diterpenoid epoxide)	*Tripterygium wilfordii*	Induces autophagy via CaMKKβ–AMPK activation and can suppress Akt/mTOR in multiple models	Highlights terpenoid control of nutrient-sensing pathways that overlap with aging mechanisms, require dose-context caution	[103]
Ginsenoside Rb1 (triterpenoid saponin)	*Panax ginseng*, *Panax notoginseng*	Modulates autophagy through AMPK-dependent mTOR signaling and autophagy regulation in vascular and renal models	Longevity relevance via vascular health and metabolic homeostasis, processes strongly tied to healthy aging	[104]
Ginkgolide B (diterpenoid lactone)	*Ginkgo biloba*	Enhances autophagy via SIRT1–FoxO1 signaling in cardiac hypertrophy models, interacts with mTOR signaling in neuronal injury contexts	Mechanistic relevance to aging heart and brain because SIRT1–FOXO and mTOR are longevity-linked nodes	[105]

Carotenoids, such as beta-carotene, lycopene, lutein, and astaxanthin, are lipid-soluble pigments found in fruits, vegetables, and marine organisms [106]. While carotenoids are primarily known for their antioxidant properties, they also influence autophagy by modulating redox-sensitive signaling pathways. By preserving redox balance, carotenoids prevent redox-mediated impairment of autophagy-related proteins and lysosomal enzymes. Emerging evidence suggests that carotenoids may also impact AMPK and TFEB activity, promoting mitochondrial turnover and cellular renewal during aging [107]. The most important carotenoids that function as autophagy modulators and are crucial to human aging biology and longevity regulation are presented in Table 5. These compounds influence redox-sensitive signaling pathways, AMPK-mTOR regulation, TFEB-associated lysosomal competence, and the restoration of autophagic flux.

Carotenoids, including β-carotene, lycopene, lutein, zeaxanthin, zeaxanthin dipalmitate, astaxanthin, fucoxanthin, β-cryptoxanthin, canthaxanthin, crocetin, and bixin, are predominantly categorized as lipophilic, chain-breaking radical-trapping antioxidants and physical quenchers of singlet oxygen [108,109]. Their conjugated polyene backbones facilitate electron transfer and radical adduct generation methods, as opposed to the usual sequential proton-loss electron transfer characteristic of phenolics [110]. The donation of hydrogen atoms is of lesser significance compared to energy transfer and radical scavenging stabilized by conjugation.

Their antioxidant activity is significantly influenced by solvent polarity and oxygen tension. Carotenoids have significant lipophilicity and are situated within lipid bilayers, mitochondria, and lipoproteins, where they effectively neutralize lipid peroxyl radicals (ROO•) and quench singlet oxygen [111]. Their activity escalates in hydrophobic membrane settings, while aqueous radical scavenging is constrained [112]. At elevated oxygen levels, several carotenoids may display pro-oxidant properties, underscoring context dependence [113]. They are very efficacious against peroxyl radicals and singlet oxygen, and they indirectly reduce the generation of superoxide and hydroxyl radicals by constraining lipid peroxidation cascades [114]. Direct reactivity with nitric oxide species is constrained, although indirect anti-nitrosative effects arise from the maintenance of membrane integrity.

Carotenoids do not directly facilitate the enzymatic decomposition of ROS/RNS but augment intrinsic defense mechanisms via Nrf2 activation and the maintenance of glutathione reserves [115]. Carotenoids play a crucial role in regenerating α-tocopherol in membranes by diminishing the tocopheroxyl radical [116]. They additionally facilitate redox cycling systems that involve glutathione and coenzyme Q10 by stabilizing lipid compartments [117]. This comprehensive chemical–physiological research elucidates that carotenoids primarily act as membrane-localized radical quenchers and redox stabilizers, with their autophagy-modulating activities intricately associated with the maintenance of lysosomal and mitochondrial integrity during aging [118].

**Table 5:** Carotenoids function as autophagy modulators critical to human aging biology and longevity regulation.

Carotenoid	Major Sources	Autophagy-Modulating Mechanism	Aging and Longevity Relevance	Ref.
β-Carotene	Carrots, sweet potato, pumpkin, leafy greens	Enhances autophagy markers and signaling consistent with ↑ AMPK, ↓ mTOR, ↑ Beclin-1/LC3-II, ↓ p62 in disease models	Supports metabolic resilience pathways that overlap with aging biology, helps maintain cellular cleanup under stress	[119]
Lycopene	Tomato, watermelon, guava, pink grapefruit	Modulates autophagy-related signaling through AMPK/mTOR (reported with downstream inflammasome control in vascular cells)	Relevant to vascular aging and inflammaging concepts via AMPK–mTOR balance and stress adaptation	[120]
Lutein	Spinach, kale, corn, egg yolk	Regulates autophagy in a context-dependent manner, including autophagy flux modulation and emerging evidence for TFEB-linked lipophagy/autophagy in liver settings	Particularly relevant to aging tissues such as retina and liver where oxidative stress and impaired autophagy contribute to functional decline	[121]
Zeaxanthin	Corn, goji berries, orange pepper, egg yolk	Primarily supports autophagy indirectly by preserving redox balance (Nrf2-linked antioxidant defense), which can prevent oxidative impairment of lysosomes and autophagy proteins	Supports “healthy aging” by limiting oxidative damage that destabilizes autophagy-lysosome function	[122]
Zeaxanthin dipalmitate	Goji (wolfberry)	Reported to restore mitochondrial autophagy functions suppressed by ethanol intoxication, with membrane receptor involvement (P2X7, AdipoR1) in liver injury models	Illustrates carotenoid support of mitophagy and organ resilience, mechanisms relevant to metabolic aging	[123]
Astaxanthin	Microalgae (*Haematococcus*), salmon, shrimp, krill	Enhances clearance of misfolded proteins by inducing autophagy in brain barrier and neuro models, intersects with oxidative stress pathways	Mechanistically relevant to brain aging and proteostasis maintenance via autophagy-supported clearance	[124]
Fucoxanthin	Brown seaweeds (*Laminaria*, *Undaria*)	Triggers AMPK-mediated autophagy with ↓ p-mTOR, increased autophagy markers under oxidative stress in hepatocytes	Relevant to longevity control via stress resistance and metabolic tissue homeostasis, especially under oxidative and inflammatory stress	[125]
β-Cryptoxanthin	Mandarin/orange citrus, papaya, persimmon	Improves age-related muscle phenotype with reduced p62 accumulation, consistent with improved autophagy-lysosome handling of cargo	Directly relevant to aging because p62 buildup and impaired autophagy contribute to sarcopenia-like decline	[126]
Canthaxanthin	Certain algae, fungi, salmonids (also used as feed-derived pigment)	Reported to alleviate cardiomyocyte senescence by regulating autophagy, with benefits shown in vitro and in aged mice	Links carotenoid-driven autophagy regulation to cardiac aging and fibrosis, key longevity determinants	[127]
Crocetin (apocarotenoid)	Saffron (*Crocus sativus*)	Induces autophagy via STK11/LKB1-mediated AMPK activation, promoting clearance of amyloid-β in neuro models	Strong mechanistic relevance to brain aging and neurodegeneration through AMPK-driven autophagic clearance	[128]
Bixin	Annatto (*Bixa orellana*)	Engages p62-dependent autophagy to regulate signaling protein turnover in fibrosis models	Supports the concept of autophagy-mediated proteostasis and inflammation control relevant to tissue aging	[129]

Bioactive natural compounds collectively modulate autophagy, reduce oxidative stress, regulate inflammation, and support mitochondrial homeostasis [130]. The ability of these compounds to restore autophagic balance, rather than causing unregulated autophagy induction, is particularly relevant for aging and lifespan control, where fine-tuned regulation is crucial. These compounds mimic aspects of caloric restriction and metabolic adaptation, offering a nutraceutical-based approach to modulating aging pathways. While challenges in bioavailability, dosage optimization, and tissue specificity remain, bioactive natural products hold promise as a foundation for developing safe and effective autophagy-targeted interventions to promote healthy human aging and longevity.

## 4. Pharmacokinetic and Pharmacodynamic Considerations of Bioactive Natural Compounds Targeting Autophagy and Mitochondrial Health in Aging

An exhaustive assessment of the drugs’ potential utility necessitates the incorporation of pharmacokinetic and pharmacodynamic factors, in conjunction with their redox chemistry and autophagy-modulating characteristics. Despite numerous polyphenols, flavonoids, terpenoids, alkaloids, and carotenoids exhibiting significant mechanistic effects in vitro and in animal models, their translational significance in aging biology is fundamentally contingent upon absorption, bioavailability, tissue distribution, metabolism, excretion, and molecular target interaction.

### 4.1. Pharmacokinetics

Polyphenols like resveratrol, quercetin, fisetin, and EGCG typically demonstrate moderate oral absorption but display low systemic bioavailability due to swift phase II metabolism, encompassing glucuronidation, sulfation, and methylation [131]. Their circulating forms are frequently conjugated metabolites rather than free aglycones, potentially affecting efficacy and cellular uptake. Lipophilic terpenoids, including ursolic acid, oleanolic acid, celastrol, and ginsenosides, have restricted water solubility and inconsistent intestinal absorption, frequently influenced by dietary lipids, micellar integration, or microbial degradation [132]. Carotenoids such as β-carotene, lycopene, lutein, and astaxanthin are extremely lipophilic and necessitate lipid-rich diets for maximum absorption; they accumulate in membranes, mitochondria, and lipoproteins, facilitating prolonged antioxidant activity while exhibiting sluggish turnover [133]. Alkaloids like berberine and caffeine are rather well absorbed; yet berberine experiences significant first-pass metabolism and displays low plasma concentrations despite its pharmacological effects, possibly due to tissue buildup and microbiome-mediated biotransformation [134]. Spermidine is effectively absorbed and widely disseminated, indicating its intrinsic biological function [135]. Mitochondrial targeting is influenced by distribution patterns. Lipophilic substances preferentially associate with lipid bilayers and mitochondrial membranes, augmenting their capacity to inhibit lipid peroxidation and maintain mitochondrial integrity [136]. Hydrophilic flavonoids may primarily reside in the cytosol unless altered to enhance membrane permeability [137]. Polyphenols are predominantly excreted through renal and biliary routes as conjugates, while carotenoids demonstrate extended half-lives owing to adipose storage [138]. These pharmacokinetic characteristics partially elucidate the discrepancy between in vitro dosage and attainable human plasma concentrations.

### 4.2. Pharmacodynamics

In addition to direct radical scavenging, most chemicals demonstrate biological effects via receptor-mediated and post-receptor signaling interactions. Polyphenols and flavonoids often activate AMPK, inhibit mTORC1, modify SIRT1, and facilitate TFEB nuclear translocation, hence improving autophagic flux and mitochondrial quality control [139]. Terpenoids like celastrol and carnosic acid may engage with thiol residues in Keap1, thereby activating Nrf2-dependent antioxidant pathways [140]. Berberine directly activates AMPK by modulating mitochondrial complex I, whereas caffeine affects Ca^2+^-AMPK signaling pathways [141]. Spermidine modifies the state of acetylation of autophagy-related proteins, signifying an epigenetic pharmacodynamic mechanism [65]. Post-receptor effects encompass phosphorylation cascades, modification of redox-sensitive cysteine residues, changes in mitochondrial membrane potential, and regulation of lysosomal activity [142]. Notably, dose-dependent hormetic responses are prevalent; moderate autophagy promotes cellular homeostasis, whereas excessive activation may lead to cytotoxicity.

The pharmacokinetic and pharmacodynamic factors collectively highlight that the ability of bioactive natural substances to modulate autophagy and mitochondria in aging cannot be determined merely by their in vitro antioxidant capability. Therapeutic significance is contingent upon tissue-specific distribution, metabolic transformation, molecular target engagement, and context-dependent signaling integration.

## 5. Plant-Derived Bioactive Natural Products in Metabolic and Lipid-Modulating Contexts Relevant to Autophagy Modulation in Human Aging

Plant-derived bioactive natural compounds significantly influence the regulation of metabolic and lipid balance; processes intricately associated with autophagy and the biology of aging [143]. Age-related metabolic dysfunction, including insulin resistance, dyslipidemia, visceral fat accumulation, and persistent low-grade inflammation, leads to compromised autophagic flux and expedited cellular senescence [144]. Recent research indicates that certain plant-derived chemicals can restore metabolic equilibrium while concurrently regulating autophagy-related signaling pathways. Polyphenols, including resveratrol, quercetin, and epigallocatechin gallate, stimulate AMPK and SIRT1 signaling while inhibiting mTORC1 activity, thus augmenting autophagic flux and boosting mitochondrial quality control [145]. Curcumin and berberine exhibit lipid-lowering effects by modulating hepatic lipid metabolism, enhancing insulin sensitivity, and reducing oxidative stress, mechanisms often associated with increased LC3-II formation and decreased p62 accumulation in experimental models [146]. Carotenoids, such as lycopene and astaxanthin, enhance redox stability and safeguard against lipid peroxidation, thereby indirectly bolstering lysosomal functionality and autophagy efficacy [147]. In human observational and interventional studies, plant-derived extracts have demonstrated enhancements in lipid profiles, inflammatory markers, and metabolic parameters related to aging [148]. Nevertheless, direct assessment of autophagy in humans is constrained, with most conclusions derived from subsequent metabolic results. These bioactive chemicals collectively serve as potential modulators of the metabolic-autophagy axis in human aging; nevertheless, long-term, biomarker-guided clinical investigations are necessary to validate their effects on lifespan.

## 6. Preclinical Findings and Human Observational or Interventional Evidence of Bioactive Natural Products in Autophagy Modulation During Human Aging

Preclinical research furnishes substantial mechanistic evidence that several bioactive natural compounds influence autophagy pathways pertinent to aging biology. In cellular and animal models, compounds such as spermidine, resveratrol, curcumin, quercetin, and epigallocatechin gallate activate AMPK, inhibit mTORC1, enhance SIRT1 signaling, and promote TFEB-mediated lysosomal biogenesis, collectively restoring autophagic flux and improving mitochondrial quality control [139,149]. These therapies often enhance metabolic balance, diminish oxidative stress, mitigate inflammation, and prolong lifespan in model species. Conversely, human evidence is still relatively scarce but is developing. Observational cohort studies indicate correlations between increased consumption of polyphenol-rich or spermidine-rich foods and decreased all-cause mortality, enhanced cardiovascular health, and reduced inflammatory burden [150]. Small-scale interventional trials have demonstrated that spermidine supplementation can enhance indicators of cognitive function and cellular aging, whereas resveratrol and polyphenol-based therapies influence metabolic parameters and inflammatory markers, aligning with autophagy-related pathways [151]. Nonetheless, direct quantification of autophagic flux in humans continues to pose significant technical challenges, leading most investigations to depend on indirect indicators [152]. These findings collectively underscore promising translational significance while emphasizing the necessity for rigorously controlled longitudinal studies to ascertain causal impacts on human lifespan.

## 7. Therapeutic and Clinical Application of Bioactive Natural Products Autophagy Modulation in Aging

**Figure 4:**
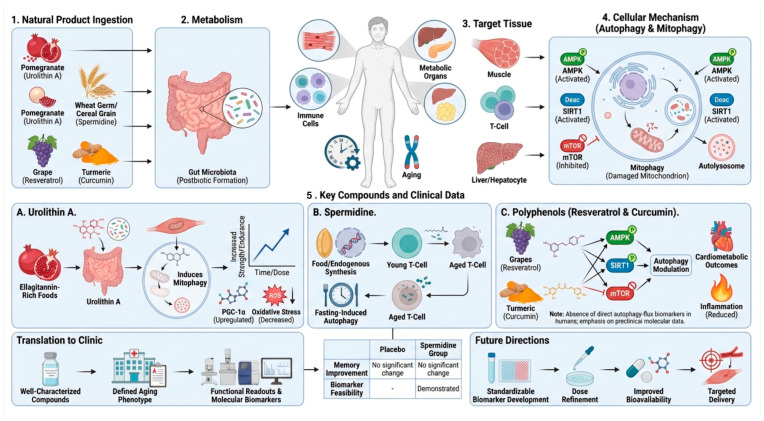
**Translational pathway of bioactive natural products in autophagy and mitophagy regulation during aging.** Natural product consumption emphasizes key sources such as pomegranate (precursors of urolithin A), wheat germ and cereal grains (spermidine), grapes (resveratrol), and turmeric (curcumin). Metabolism highlights the essential function of gut microbiota in transforming food precursors into bioactive metabolites, especially postbiotic molecules like urolithin A. Target tissues encompass metabolically active and aging-sensitive systems, including skeletal muscle, immunological cells (particularly T cells), and the liver, where a loss in autophagy contributes to functional degradation. Cellular mechanisms illustrate the activation of AMPK and SIRT1 signaling, along with the inhibition of mTOR, resulting in the induction of autophagy and selective mitophagy, autophagosome formation, and autolysosomal destruction of impaired mitochondria. The lower panels encapsulate essential translational examples: (**A**) Urolithin A promotes mitophagy, augments PGC-1α signaling, diminishes oxidative stress, and enhances muscle endurance in a dose- and time-dependent fashion. (**B**) Spermidine facilitates fasting-induced autophagy and maintains autophagic efficacy in aging T cells. (**C**) Polyphenols, including resveratrol and curcumin, influence AMPK-SIRT1-mTOR signaling to regulate autophagy, thereby contributing to cardiometabolic protection and the attenuation of inflammation.

Translation of autophagy-modulating natural products into the clinic is now moving from mechanistic rationalization to measurable human phenotypes, but it has been uneven. The most “clinically mature” cases are where the therapies enhance mitochondrial quality control or restore autophagic function in aging-related tissues such as skeletal muscle, immune cells, and metabolic organs. One well-studied example is urolithin A, a gut-produced postbiotic from ellagitannin-rich foods like pomegranate. Human studies have shown that it is safe and can produce a molecular profile that is consistent with improved mitochondrial and cellular health commensurate with its role as a mitophagy inducer. This provides a relatively pragmatic translational approach: use a well-characterized compound, target a defined aging phenotype (mitophagy), and then measure functional readouts (strength, endurance) with molecular biomarkers (Figure 4).

Clinical studies often use surrogate outcomes like inflammation or metabolic markers, while direct measurement of autophagic flux in humans is limited by the lack of standardized, non-invasive biomarkers. Natural products are chemically heterogeneous and can vary between preparations, posing reproducibility and standardization challenges. Aging is highly heterogeneous between individuals, and comorbidities, polypharmacy, and sex-specific factors can confound interpretation and limit generalizability.

Another clinically relevant axis is spermidine, a supplemental autophagy inducer that declines in human T cells with age. A long-term randomized trial in older individuals at risk of Alzheimer’s disease did not improve memory but did provide clarity on feasibility and biomarker approaches for future studies. Spermidine is mechanistically linked to fasting-mediated autophagy, and the data from human subjects is consistent with an in vivo role. The clinical use of more traditional polyphenols like resveratrol and curcumin has been focused on cardiometabolic and inflammatory endpoints, but direct autophagy-flux biomarkers in humans are rare. The strongest data set here remains at the molecular and preclinical level with respect to AMPK, SIRT1, and mTOR-related autophagy regulation. Current uses are moving towards using standardizable biomarkers, dose refinement, and formulations with better bioavailability and tissue targeting. Table 6 presents clinical and translational examples of natural compounds that influence autophagy or mitophagy in the context of aging.

**Table 6:** Therapeutic and clinical applications of bioactive natural products in the control of autophagy related to aging.

Bioactive Natural Product	Human Use Case (Examples)	Autophagy or Mitophagy Mechanism (Key Nodes)	Human Evidence, Endpoints	Ref.
Urolithin A	Healthy aging, muscle function	Mitophagy activation improves mitochondrial quality signatures	Reported safety in humans and induces molecular signatures of improved mitochondrial and cellular health, trials report improvements in muscle performance measures	[153]
Spermidine	Aging biology, cognition-focused trials	Autophagy induction via epigenetic regulation, supports fasting-mediated autophagy	Trial in older adults at risk for Alzheimer disease did not improve memory, informs feasibility and biomarker selection	[154]
Spermidine	Immune aging (T-cell function)	Age-related decline in spermidine associates with reduced autophagy capacity in human T cells	Mechanistic human evidence linking spermidine levels and autophagy in T cells	[155]
Resveratrol	Cardiometabolic and vascular aging research	Autophagy induction via mTOR–ULK1 inhibition, AMPK and SIRT1 linkage	Mostly mechanistic and preclinical support for autophagy targeting clinical endpoints often indirect	[156]
Curcumin	Inflammation and oxidative stress settings	Cytoprotective autophagy under oxidative stress, autophagy-linked protection in endothelial cells	Clinical use largely targets inflammation, direct human autophagy-flux readouts are still uncommon	[157]
EGCG (green tea catechin)	Vascular and metabolic protection concepts	Stimulates autophagic flux, increases lysosomal acidification in experimental systems	Strong mechanistic basis, clinical translation mostly indirect, needs standardized human autophagy biomarkers	[158]

## 8. Translational Limitations of Bioactive Natural Products Targeting Autophagy in Human Aging

Despite strong mechanistic evidence from cellular and animal models, substantial translational obstacles hinder the therapeutic use of bioactive natural compounds as autophagy modulators in human aging. Bioavailability is a big problem. A lot of polyphenols and related compounds, such as resveratrol and curcumin, do not dissolve well, break down quickly, or get into the body very easily, which makes it hard to get concentrations that are like those employed in experimental settings [159]. The amount of the drug is still a big problem. In vitro, effective concentrations are often beyond levels attainable by diet alone, and consistent dosage regimens in humans are not yet well established. Moreover, the long-term safety profile of persistent autophagy regulation is not yet fully understood. Moderate activation of autophagy may facilitate cellular homeostasis; however, excessive or prolonged activation could potentially lead to muscular atrophy, disrupted immunological equilibrium, or unexpected metabolic consequences, especially in older adults [160]. Another significant factor is inter-individual variability. Genetic background, epigenetic status, gut microbiome makeup, nutritional condition, comorbidities, and sex-specific variations all affect how well autophagy works [161]. Aging modifies pharmacokinetics and stress signaling pathways, challenging the extrapolation from controlled models to diverse human populations. To overcome these translational challenges, we will need standardized formulations, dosage regimens based on biomarkers, and well-planned, long-term clinical trials that target different groups of older people.

## 9. Limitations and Future Direction of Bioactive Natural Products in Autophagy Modulation in Aging

There are multiple limitations in translating the strong molecular relationships between bioactive natural products and autophagy regulation to clinical aging. Drugs have poor oral bioavailability, rapid metabolism, and inter-individual variability related to alterations in gut microbiota, which can lead to inconsistent tissue exposure and variable responses. Autophagy is highly context-dependent and too much or too little autophagy can be detrimental, and as such, the dosage, timing, and tissue specificity of these interventions are key, but are often not well-defined.

For future progress, robust translation strategies will be needed to bridge autophagy modulation and quantifiable clinical endpoints. The development of standardized biomarkers for human autophagy and lysosomal function, including blood-based signatures, imaging modalities, and validated surrogate markers will help improve the interpretability of studies. Improved formulations, including nanoparticle delivery systems, prodrugs, and microbiome-informed postbiotics, could improve bioavailability and allow for tissue-specific targeting while reducing off-target effects. Personalized approaches will be needed to stratify subjects based on metabolic state, inflammatory burden, microbiome composition, and genetic variability in autophagy pathways to identify responders and optimize dosing regimens. Synergistic interventions that combine natural products with lifestyle interventions like exercise, time-restricted feeding, or protein periodization may produce additive effects on AMPK, SIRT1, TFEB, and mitophagy pathways relevant to healthy aging. In the long term, large-scale randomized controlled trials with standardized endpoints and long-term safety evaluations will be essential to move autophagy-targeting natural products from promising candidates to evidence-based interventions for aging.

## 10. Conclusions

Autophagy is a pivotal regulatory process that connects cellular quality control with aging and longevity, and its gradual decline considerably contributes to age-related functional decline and disease vulnerability. Increasing data indicates that bioactive natural compounds, including polyphenols, flavonoids, alkaloids, terpenoids, and carotenoids, can regulate autophagy in a nuanced and context-sensitive manner. Instead of serving as indiscriminate autophagy inducers, these drugs precisely modulate critical signaling pathways, including AMPK, PI3K/AKT/mTOR, SIRT1, FOXO, and TFEB, while concurrently diminishing oxidative stress and chronic inflammation that hinder autophagic flux in aging. These coordinated effects enable natural products to promote proteostasis, mitochondrial quality control, and metabolic flexibility, which are crucial for sustaining tissue homeostasis and promoting healthy longevity. Despite ongoing obstacles concerning bioavailability, inter-individual variability, and the validation of clinical biomarkers, advancements in formulation, precision nutrition, and trial design are swiftly enhancing translational potential. Autophagy-targeting bioactive natural compounds collectively constitute a promising, mechanistically sound approach for enhancing healthy human aging and prolonging health span.

## Data Availability

No new data were created or analyzed in this study.
